# Supplementary material for: Estimating road traffic impacts of commute mode shifts
Source: PLoS One. 2023 Jan 11;18(1):e0279738. doi: 10.1371/journal.pone.0279738 (PMC9833534; doi:10.1371/journal.pone.0279738)
Supplement: S1 Table — (PDF) [file pone.0279738.s001.pdf]

Table S1. Common shorthand Metropolitan Statistical Area (MSA) name and the corresponding complete US Census Bureau (USCB) Official Metropolitan Statistical Area name.

MSA shorthand	Complete USCB MSA name
Akron, OH	Akron, OH Metro Area
Albany, NY	Albany-Schenectady-Troy, NY Metro Area
Albuquerque, NM	Albuquerque, NM Metro Area
Allentown, PA	Allentown-Bethlehem-Easton, PA-NJ Metro Area
Anchorage, AK	Anchorage, AK Metro Area
Ann Arbor, MI	Ann Arbor, MI Metro Area
Atlanta, GA	Atlanta-Sandy Springs-Roswell, GA Metro Area
Atlantic City, NJ	Atlantic City-Hammonton, NJ Metro Area
Austin, TX	Austin-Round Rock, TX Metro Area
Bakersfield, CA	Bakersfield, CA Metro Area
Baltimore, MD	Baltimore-Columbia-Towson, MD Metro Area
Baton Rouge, LA	Baton Rouge, LA Metro Area
Birmingham, AL	Birmingham-Hoover, AL Metro Area
Boise City, ID	Boise City, ID Metro Area
Boston, MA	Boston-Cambridge-Newton, MA-NH Metro Area
Boulder, CO	Boulder, CO Metro Area
Bremerton, WA	Bremerton-Silverdale, WA Metro Area
Bridgeport, CT	Bridgeport-Stamford-Norwalk, CT Metro Area
Buffalo, NY	Buffalo-Cheektowaga-Niagara Falls, NY Metro Area
Burlington, VT	Burlington-South Burlington, VT Metro Area
Cape Coral, FL	Cape Coral-Fort Myers, FL Metro Area
Champaign, IL	Champaign-Urbana, IL Metro Area
Charleston, SC	Charleston-North Charleston, SC Metro Area
Charlotte, NC	Charlotte-Concord-Gastonia, NC-SC Metro Area
Chattanooga, TN	Chattanooga, TN-GA Metro Area
Chicago, IL	Chicago-Naperville-Elgin, IL-IN-WI Metro Area
Cincinnati, OH	Cincinnati, OH-KY-IN Metro Area
Cleveland, OH	Cleveland-Elyria, OH Metro Area
Colorado Springs, CO	Colorado Springs, CO Metro Area
Columbia, SC	Columbia, SC Metro Area
Columbus, OH	Columbus, OH Metro Area
Dallas, TX	Dallas-Fort Worth-Arlington, TX Metro Area
Davenport, IA	Davenport-Moline-Rock Island, IA-IL Metro Area
Dayton, OH	Dayton, OH Metro Area
Deltona, FL	Deltona-Daytona Beach-Ormond Beach, FL Metro Area
Denver, CO	Denver-Aurora-Lakewood, CO Metro Area
Des Moines, IA	Des Moines-West Des Moines, IA Metro Area
Detroit, MI	Detroit-Warren-Dearborn, MI Metro Area
Duluth, MN	Duluth, MN-WI Metro Area
Durham, NC	Durham-Chapel Hill, NC Metro Area
El Paso, TX	El Paso, TX Metro Area
Eugene, OR	Eugene, OR Metro Area
Fort Collins, CO	Fort Collins, CO Metro Area
Fort Wayne, IN	Fort Wayne, IN Metro Area
Fresno, CA	Fresno, CA Metro Area
Grand Rapids, MI	Grand Rapids-Wyoming, MI Metro Area
Greensboro, NC	Greensboro-High Point, NC Metro Area
Greenville, SC	Greenville-Anderson-Mauldin, SC Metro Area
Harrisburg, PA	Harrisburg-Carlisle, PA Metro Area
Hartford, CT	Hartford-West Hartford-East Hartford, CT Metro Area

Continued on the next page

Table S1 – continued from previous page

MSA shorthand	USCB MSA name
Honolulu, KY	Urban Honolulu, HI Metro Area
Houston, TX	Houston-The Woodlands-Sugar Land, TX Metro Area
Indianapolis, IN	Indianapolis-Carmel-Anderson, IN Metro Area
Jacksonville, FL	Jacksonville, FL Metro Area
Kansas City, MO	Kansas City, MO-KS Metro Area
Knoxville, TN	Knoxville, TN Metro Area
Lancaster, PA	Lancaster, PA Metro Area
Lansing, MI	Lansing-East Lansing, MI Metro Area
Las Vegas, NV	Las Vegas-Henderson-Paradise, NV Metro Area
Lexington, KY	Lexington-Fayette, KY Metro Area
Lincoln, NE	Lincoln, NE Metro Area
Little Rock, AR	Little Rock-North Little Rock-Conway, AR Metro Area
Los Angeles, CA	Los Angeles-Long Beach-Anaheim, CA Metro Area
Louisville, KY	Louisville/Jefferson County, KY-IN Metro Area
Madison, WI	Madison, WI Metro Area
Manchester, NH	Manchester-Nashua, NH Metro Area
McAllen, TX	McAllen-Edinburg-Mission, TX Metro Area
Memphis, TN	Memphis, TN-MS-AR Metro Area
Miami, FL	Miami-Fort Lauderdale-West Palm Beach, FL Metro Area
Milwaukee, WI	Milwaukee-Waukesha-West Allis, WI Metro Area
Minneapolis, MN	Minneapolis-St. Paul-Bloomington, MN-WI Metro Area
Nashville, TN	Nashville-Davidson–Murfreesboro–Franklin, TN Metro Area
New Haven, CT	New Haven-Milford, CT Metro Area
New Orleans, LA	New Orleans-Metairie, LA Metro Area
New York, NY	New York-Newark-Jersey City, NY-NJ-PA Metro Area
North Port, FL	North Port-Sarasota-Bradenton, FL Metro Area
Ogden, UT	Ogden-Clearfield, UT Metro Area
Oklahoma City, OK	Oklahoma City, OK Metro Area
Omaha, NE	Omaha-Council Bluffs, NE-IA Metro Area
Orlando, FL	Orlando-Kissimmee-Sanford, FL Metro Area
Oxnard, CA	Oxnard-Thousand Oaks-Ventura, CA Metro Area
Peoria, IL	Peoria, IL Metro Area
Philadelphia, PA	Philadelphia-Camden-Wilmington, PA-NJ-DE-MD Metro Area
Phoenix, AZ	Phoenix-Mesa-Scottsdale, AZ Metro Area
Pittsburgh, PA	Pittsburgh, PA Metro Area
Portland, ME	Portland-South Portland, ME Metro Area
Portland, OR	Portland-Vancouver-Hillsboro, OR-WA Metro Area
Providence, RI	Providence-Warwick, RI-MA Metro Area
Provo, UT	Provo-Orem, UT Metro Area
Raleigh, NC	Raleigh, NC Metro Area
Reading, PA	Reading, PA Metro Area
Reno, NV	Reno, NV Metro Area
Richmond, VA	Richmond, VA Metro Area
Riverside, CA	Riverside-San Bernardino-Ontario, CA Metro Area
Rochester, MN	Rochester, MN Metro Area
Rochester, NY	Rochester, NY Metro Area
Sacramento, CA	Sacramento–Roseville–Arden-Arcade, CA Metro Area
Salem, OR	Salem, OR Metro Area
Salinas, CA	Salinas, CA Metro Area
Salt Lake City, UT	Salt Lake City, UT Metro Area
San Antonio, TX	San Antonio-New Braunfels, TX Metro Area
San Diego, CA	San Diego-Carlsbad, CA Metro Area

Continued on the next page

Table S1 – continued from previous page

MSA shorthand	USCB MSA name
San Francisco, CA	San Francisco-Oakland-Hayward, CA Metro Area
San Jose, CA	San Jose-Sunnyvale-Santa Clara, CA Metro Area
San Juan, PR	San Juan-Carolina-Caguas, PR Metro Area
Santa Cruz, CA	Santa Cruz-Watsonville, CA Metro Area
Santa Maria, CA	Santa Maria-Santa Barbara, CA Metro Area
Santa Rosa, CA	Santa Rosa, CA Metro Area
Savannah, GA	Savannah, GA Metro Area
Scranton, PA	Scranton–Wilkes-Barre–Hazleton, PA Metro Area
Seattle, WA	Seattle-Tacoma-Bellevue, WA Metro Area
South Bend, IN	South Bend-Mishawaka, IN-MI Metro Area
Spokane, WA	Spokane-Spokane Valley, WA Metro Area
Springfield, MA	Springfield, MA Metro Area
St. Louis, MO	St. Louis, MO-IL Metro Area
Stockton, CA	Stockton-Lodi, CA Metro Area
Syracuse, NY	Syracuse, NY Metro Area
Tampa, FL	Tampa-St. Petersburg-Clearwater, FL Metro Area
Toledo, OH	Toledo, OH Metro Area
Trenton, NJ	Trenton, NJ Metro Area
Tucson, AZ	Tucson, AZ Metro Area
Tulsa, OK	Tulsa, OK Metro Area
Vallejo, CA	Vallejo-Fairfield, CA Metro Area
Virginia Beach, VA	Virginia Beach-Norfolk-Newport News, VA-NC Metro Area
Visalia, CA	Visalia-Porterville, CA Metro Area
Washington, DC	Washington-Arlington-Alexandria, DC-VA-MD-WV Metro Area
Wichita, KS	Wichita, KS Metro Area
Winston, NC	Winston-Salem, NC Metro Area
Worcester, MA	Worcester, MA-CT Metro Area
York, PA	York-Hanover, PA Metro Area
Youngstown, OH	Youngstown-Warren-Boardman, OH-PA Metro Area
