# Supplementary material for: Estimating road traffic impacts of commute mode shifts
Source: PLoS One. 2023 Jan 11;18(1):e0279738. doi: 10.1371/journal.pone.0279738 (PMC9833534; doi:10.1371/journal.pone.0279738)
Supplement: S2 Table — Years of data available for fitting the model and performance measures for the fitted model. (PDF) [file pone.0279738.s002.pdf]

Table S2. Summary of BPR models for all 74 analysed metro areas. Years of data available for fitting the model and performance measures for the fitted model.

	years of data	LOO RMSE (min)	R^2
New York	6	0.48	0.73
San Francisco	9	0.73	0.92
Los Angeles	6	0.38	0.95
Boston	9	0.42	0.88
Chicago	9	0.30	0.78
Philadelphia	9	0.25	0.93
Seattle	9	0.32	0.97
Houston	9	0.52	0.86
Dallas	9	0.36	0.91
San Jose	9	0.93	0.87
Atlanta	9	0.38	0.92
Miami	9	0.37	0.94
Portland	9	0.36	0.95
Riverside	8	0.40	0.93
Orlando	9	0.60	0.85
Washington	9	0.48	0.42
Baltimore	9	0.40	0.80
Tampa	9	0.25	0.94
Denver	9	0.17	0.97
Providence	9	0.48	0.73
Jacksonville	9	0.66	0.73
San Diego	9	0.34	0.93
Phoenix	9	0.27	0.77
San Antonio	9	0.46	0.74
Cincinnati	9	0.27	0.71
Oxnard	9	0.59	0.88
Raleigh	9	0.33	0.94
Austin	6	0.14	0.98
St. Louis	9	0.33	0.79
Charlotte	6	0.15	0.97
Pittsburgh	9	0.34	0.52
North Port	8	0.87	0.72
Allentown	9	0.58	0.63
Oklahoma City	9	0.22	0.88
Nashville	9	0.24	0.96
Minneapolis	9	0.14	0.79
Charleston	6	0.71	0.88
Sacramento	9	0.29	0.91
Santa Rosa	9	0.77	0.55
Boise City	9	0.54	0.61
Kansas City	9	0.21	0.81
Louisville	9	0.29	0.50
Savannah	6	0.69	0.74
Reading	9	0.57	0.44
Bremerton	8	0.96	0.51
Richmond	9	0.34	0.67
Bridgeport	9	0.66	0.43
Buffalo	9	0.44	0.47
Boulder	9	0.84	0.54

Continued on the next page

Table S2 – continued from previous page

	years of data	LOO RMSE (min)	R^2
Lexington	7	0.68	0.48
Ann Arbor	9	0.37	0.73
Tucson	9	0.57	0.55
Durham	9	0.31	0.91
Omaha	9	0.24	0.62
Hartford	9	0.45	0.45
Lancaster	9	0.67	0.74
Colorado Springs	9	0.53	0.36
Lincoln	9	0.71	0.51
Salinas	9	0.55	0.42
Rochester	9	0.68	0.42
Duluth	9	0.66	0.34
Provo	9	0.65	0.49
Salt Lake City	9	0.37	0.64
Greenville	6	0.42	0.80
Memphis	9	0.21	0.61
Stockton	9	1.00	0.86
Vallejo	9	0.99	0.58
Las Vegas	9	0.40	0.69
Ogden	9	0.31	0.62
Virginia Beach	9	0.47	0.48
Fresno	9	0.71	0.35
Baton Rouge	9	0.86	0.49
Detroit	9	0.30	0.78
Tulsa	9	0.53	0.36
