# Supplementary material for: Estimating road traffic impacts of commute mode shifts
Source: PLoS One. 2023 Jan 11;18(1):e0279738. doi: 10.1371/journal.pone.0279738 (PMC9833534; doi:10.1371/journal.pone.0279738)
Supplement: S3 Table — All 74 analysed cites are shown, ranked by total cost per day. The range shows one standard deviation of the predictions. M denotes millions. B denotes billions. The major city is listed here in representation of the metro area. (PDF) [file pone.0279738.s003.pdf]

Table S3. Summary of city transportation status in 2018 and prediction if one in four commuters switch from transit or car share mode to SOV mode. All 74 analysed cites are shown, ranked by total cost per day. The range shows one standard deviation of the predictions. M denotes millions. B denotes billions. The major city is listed here in representation of the metro area.

	transportation status in 2018				prediction for 25% switch			
	total commuters (M)	passenger vehicles (M)	transit riders (M) & (% of total commuters)	travel time (min)	passenger vehicles (M)	added time (min)	added $ cost per person	total added $ cost per year (B)
New York	8.72	5.16	3.0(34.43%)	31.00	6.05	6.7±0.6	1065.0±95.0	25.78±8.65
San Francisco	2.14	1.49	0.42(19.56%)	34.30	1.65	10.0±0.8	1601.0±128.0	10.58±1.85
Los Angeles	5.92	5.13	0.31(5.24%)	31.60	5.32	1.4±0.4	220.0±63.0	4.69±1.62
Boston	2.30	1.79	0.34(14.90%)	31.70	1.92	3.0±0.6	470.0±102.0	3.61±0.98
Chicago	4.32	3.46	0.57(13.19%)	30.10	3.68	1.4±0.2	216.0±30.0	3.17±1.29
Philadelphia	2.71	2.25	0.29(10.78%)	28.80	2.36	1.8±1.1	291.0±178.0	2.75±0.65
Seattle	1.84	1.46	0.22(11.95%)	30.60	1.55	2.6±0.6	422.0±100.0	2.62±0.48
Houston	3.10	2.81	0.06(2.09%)	30.40	2.88	0.5±0.3	87.0±44.0	1.0±1.15
Dallas	3.49	3.20	0.05(1.42%)	28.80	3.27	0.4±0.5	60.0±77.0	0.78±0.83
San Jose	0.95	0.82	0.04(4.26%)	33.00	0.85	2.2±0.5	344.0±84.0	1.17±0.79
Atlanta	2.68	2.39	0.09(3.27%)	32.40	2.46	0.7±1.1	107.0±173.0	1.05±0.8
Miami	2.77	2.44	0.09(3.38%)	29.90	2.53	0.9±0.5	149.0±81.0	1.5±0.85
Portland	1.12	0.95	0.08(6.92%)	27.00	0.99	1.2±0.4	195.0±58.0	0.77±0.28
Riverside	1.86	1.69	0.02(1.33%)	27.80	1.73	0.5±0.2	80.0±31.0	0.55±0.5
Orlando	1.17	1.06	0.02(1.43%)	29.90	1.09	0.5±0.7	84.0±111.0	0.37±0.48
Washington	3.03	2.34	0.44(14.36%)	34.30	2.52	0.6±0.4	101.0±59.0	1.02±1.18
Baltimore	1.30	1.14	0.08(6.49%)	29.90	1.18	1.1±0.8	171.0±129.0	0.81±0.45
Tampa	1.31	1.20	0.02(1.45%)	27.60	1.23	0.5±1.1	73.0±180.0	0.36±0.25
Denver	1.43	1.27	0.06(4.26%)	27.60	1.31	0.5±0.3	79.0±42.0	0.41±0.18
Providence	0.75	0.68	0.02(2.52%)	23.70	0.70	0.6±0.9	90.0±149.0	0.25±0.29
Jacksonville	0.67	0.61	0.01(1.03%)	27.30	0.63	0.4±0.3	69.0±48.0	0.17±0.32
San Diego	1.49	1.33	0.04(2.93%)	26.90	1.37	0.8±0.8	122.0±127.0	0.67±0.4
Phoenix	2.07	1.84	0.04(1.96%)	26.10	1.90	0.3±0.6	41.0±101.0	0.31±0.44
San Antonio	1.08	0.97	0.02(1.90%)	25.90	1.00	0.4±1.1	66.0±169.0	0.26±0.4
Cincinnati	1.00	0.92	0.02(1.83%)	25.20	0.94	0.3±0.8	47.0±122.0	0.18±0.22
Oxnard	0.38	0.35	0.0(1.15%)	24.80	0.36	1.0±0.7	151.0±110.0	0.22±0.18
Raleigh	0.63	0.59	0.01(1.02%)	26.80	0.60	0.4±0.6	60.0±99.0	0.14±0.14
Austin	1.03	0.93	0.02(2.08%)	28.00	0.96	0.4±0.7	69.0±111.0	0.26±0.12
St. Louis	1.30	1.21	0.03(2.19%)	25.90	1.23	0.5±0.4	76.0±55.0	0.38±0.33
Charlotte	1.17	1.07	0.02(1.67%)	27.40	1.10	0.3±0.4	52.0±71.0	0.23±0.12
Pittsburgh	1.05	0.92	0.06(6.10%)	26.60	0.95	0.6±0.2	89.0±39.0	0.34±0.32
North Port	0.31	0.29	0.0(0.59%)	25.60	0.29	0.5±0.4	78.0±71.0	0.09±0.21
Allentown	0.38	0.35	0.01(1.87%)	24.40	0.35	0.5±0.4	73.0±60.0	0.1±0.14
Oklahoma City	0.64	0.59	0.0(0.67%)	23.40	0.60	0.2±0.5	39.0±75.0	0.09±0.1
Nashville	0.92	0.85	0.01(0.88%)	29.10	0.87	0.3±0.2	54.0±35.0	0.19±0.17
Minneapolis	1.79	1.58	0.09(4.90%)	25.40	1.64	0.2±0.5	32.0±80.0	0.21±0.21
Charleston	0.36	0.33	0.0(0.88%)	28.40	0.34	0.7±0.4	106.0±58.0	0.14±0.18
Sacramento	0.97	0.87	0.02(2.48%)	26.60	0.89	0.5±0.7	73.0±113.0	0.26±0.2
Santa Rosa	0.23	0.21	0.01(2.30%)	23.60	0.21	0.5±0.8	83.0±127.0	0.07±0.15
Boise City	0.32	0.29	0.0(0.30%)	22.10	0.30	0.2±0.7	27.0±107.0	0.03±0.12
Kansas City	1.01	0.94	0.01(0.97%)	23.60	0.96	0.2±0.4	30.0±72.0	0.12±0.15
Louisville	0.59	0.54	0.01(1.83%)	24.80	0.55	0.3±0.7	46.0±117.0	0.1±0.16
Savannah	0.18	0.16	0.0(1.25%)	26.70	0.17	0.4±0.8	65.0±134.0	0.04±0.08

Continued on the next page

	transportation status in 2018				prediction for 25% switch			
	total commuters (M)	passenger vehicles (M)	transit riders (M)	travel time (min)	passenger vehicles (M)	added time (min)	added $ cost per person	total added $ cost per year (B)
Reading	0.19	0.17	0.0(2.12%)	23.10	0.18	0.5±0.4	78.0±68.0	0.05±0.09
Bremerton	0.12	0.10	0.01(8.39%)	25.30	0.10	0.9±0.7	138.0±105.0	0.06±0.09
Richmond	0.61	0.55	0.01(1.82%)	25.50	0.57	0.3±0.5	54.0±74.0	0.12±0.16
Bridgeport	0.43	0.36	0.04(10.40%)	26.60	0.38	0.9±0.5	141.0±76.0	0.21±0.23
Buffalo	0.50	0.46	0.02(3.29%)	21.30	0.47	0.3±0.8	52.0±127.0	0.1±0.15
Boulder	0.15	0.12	0.01(5.99%)	26.20	0.13	0.9±1.8	150.0±280.0	0.08±0.1
Lexington	0.24	0.22	0.0(1.42%)	24.80	0.22	0.4±0.8	59.0±124.0	0.05±0.13
Ann Arbor	0.16	0.14	0.01(6.37%)	27.60	0.14	0.6±0.6	96.0±100.0	0.06±0.05
Tucson	0.42	0.37	0.01(2.32%)	24.70	0.38	0.5±0.8	83.0±126.0	0.13±0.17
Durham	0.26	0.23	0.01(3.75%)	26.90	0.24	0.5±0.4	75.0±72.0	0.07±0.06
Omaha	0.45	0.42	0.0(0.74%)	20.40	0.43	0.1±0.4	23.0±73.0	0.04±0.08
Hartford	0.56	0.52	0.02(2.83%)	24.90	0.53	0.4±0.3	69.0±45.0	0.15±0.23
Lancaster	0.24	0.21	0.0(1.03%)	21.80	0.22	0.8±0.5	130.0±82.0	0.12±0.09
Colorado Springs	0.33	0.30	0.0(0.66%)	22.80	0.31	0.1±1.0	22.0±151.0	0.03±0.13
Lincoln	0.17	0.15	0.0(1.12%)	20.20	0.16	0.2±1.2	40.0±189.0	0.03±0.08
Salinas	0.18	0.14	0.0(1.46%)	23.40	0.15	0.5±0.4	77.0±56.0	0.05±0.07
Rochester	0.11	0.09	0.01(5.09%)	20.70	0.10	0.4±0.6	67.0±88.0	0.03±0.05
Duluth	0.12	0.11	0.0(2.28%)	21.30	0.12	0.4±0.4	58.0±58.0	0.03±0.06
Provo	0.26	0.23	0.01(2.28%)	19.60	0.24	0.2±0.6	40.0±92.0	0.04±0.12
Salt Lake City	0.57	0.50	0.02(3.46%)	23.90	0.52	0.3±0.6	54.0±104.0	0.11±0.15
Greenville	0.40	0.37	0.0(0.36%)	24.10	0.38	0.3±0.8	52.0±136.0	0.08±0.12
Memphis	0.60	0.56	0.0(0.68%)	24.80	0.57	0.1±1.4	19.0±231.0	0.04±0.1
Stockton	0.30	0.27	0.01(1.67%)	27.30	0.28	0.8±1.0	133.0±163.0	0.15±0.18
Vallejo	0.20	0.17	0.01(3.54%)	25.00	0.18	0.7±0.5	109.0±82.0	0.08±0.13
Las Vegas	0.99	0.87	0.03(3.46%)	23.80	0.90	0.3±0.5	50.0±84.0	0.18±0.27
Ogden	0.30	0.27	0.0(1.58%)	19.30	0.28	0.1±0.4	19.0±69.0	0.02±0.06
Virginia Beach	0.80	0.73	0.01(1.55%)	24.90	0.75	0.4±1.4	57.0±222.0	0.17±0.27
Fresno	0.38	0.34	0.0(1.12%)	22.30	0.35	0.2±0.2	30.0±28.0	0.04±0.17
Baton Rouge	0.36	0.34	0.0(0.94%)	27.80	0.34	0.6±2.2	93.0±357.0	0.13±0.23
Detroit	1.90	1.75	0.03(1.40%)	27.40	1.79	0.2±0.8	39.0±121.0	0.28±0.42
Tulsa	0.43	0.40	0.0(0.55%)	22.10	0.41	0.2±0.3	33.0±51.0	0.05±0.17
