# Supplementary material for: Estimating road traffic impacts of commute mode shifts
Source: PLoS One. 2023 Jan 11;18(1):e0279738. doi: 10.1371/journal.pone.0279738 (PMC9833534; doi:10.1371/journal.pone.0279738)
Supplement: S4 Table — (PDF) [file pone.0279738.s004.pdf]

Table S4. Summary of inferred BPR model parameters for all 74 modeled metro areas.

	capacity (M)	free flow travel time (min)	capacity ratio 2018	travel time ratio 2018
New York	4.27	23.5	1.21	1.34
San Francisco	0.86	14.7	1.73	2.33
Los Angeles	4.10	23.1	1.25	1.37
Boston	1.39	22.3	1.29	1.44
Philadelphia	1.75	20.5	1.28	1.41
Chicago	3.24	25.1	1.07	1.21
Seattle	1.12	21.3	1.30	1.44
Houston	2.62	25.3	1.07	1.18
Dallas	3.16	24.8	1.01	1.14
San Jose	0.57	20.3	1.43	1.61
Atlanta	2.23	27.0	1.07	1.20
Miami	2.08	23.2	1.17	1.29
Portland	0.80	20.9	1.18	1.27
Riverside	1.56	23.1	1.08	1.19
Orlando	0.99	25.0	1.07	1.18
Washington	2.95	32.4	0.79	1.07
Baltimore	0.95	22.9	1.20	1.32
Tampa	1.12	23.2	1.06	1.18
Denver	1.26	24.0	1.00	1.15
Providence	0.56	17.9	1.21	1.31
Jacksonville	0.57	22.8	1.07	1.17
San Diego	1.13	20.9	1.18	1.31
Phoenix	2.15	24.2	0.85	1.08
San Antonio	0.96	22.3	1.02	1.16
Cincinnati	0.92	21.9	1.01	1.15
Oxnard	0.24	15.3	1.43	1.61
Raleigh	0.54	22.2	1.08	1.20
Austin	0.91	24.0	1.02	1.17
St. Louis	1.02	20.0	1.19	1.31
Charlotte	1.09	24.1	0.98	1.14
Pittsburgh	0.89	22.7	1.03	1.18
North Port	0.25	20.3	1.15	1.24
Allentown	0.31	19.8	1.12	1.22
Oklahoma City	0.59	20.3	1.01	1.15
Nashville	0.84	25.2	1.01	1.16
Minneapolis	1.99	24.0	0.80	1.06
Charleston	0.26	20.4	1.27	1.40
Sacramento	0.86	23.0	1.01	1.17
Boise City	0.32	20.0	0.91	1.09
Santa Rosa	0.18	19.0	1.12	1.23
Kansas City	1.00	21.1	0.95	1.12
Savannah	0.16	22.7	1.04	1.16
Louisville	0.55	21.6	0.99	1.15
Reading	0.15	18.8	1.11	1.22
Bremerton	0.09	21.2	1.06	1.18
Richmond	0.55	22.1	1.00	1.16
Bridgeport	0.34	22.6	1.04	1.20
Buffalo	0.44	18.0	1.05	1.19
Boulder	0.11	21.8	1.08	1.22
Ann Arbor	0.14	24.0	1.00	1.16

Continued on the next page

Table S4 – continued from previous page

	capacity (M)	free flow travel time (min)	capacity ratio 2018	travel time ratio 2018
Lexington	0.21	20.7	1.07	1.20
Omaha	0.46	18.5	0.91	1.11
Tucson	0.35	21.1	1.03	1.19
Durham	0.23	23.4	0.99	1.16
Colorado Springs	0.36	21.2	0.83	1.07
Lancaster	0.17	15.5	1.28	1.44
Hartford	0.47	20.4	1.10	1.24
Lincoln	0.15	17.2	1.03	1.18
Salinas	0.16	21.5	0.88	1.11
Rochester	0.09	18.1	0.99	1.17
Duluth	0.10	17.6	1.09	1.24
Salt Lake City	0.54	21.6	0.92	1.12
Provo	0.25	17.7	0.92	1.12
Greenville	0.34	20.1	1.07	1.22
Memphis	0.67	23.2	0.83	1.08
Stockton	0.22	20.0	1.25	1.41
Vallejo	0.16	20.7	1.08	1.25
Las Vegas	0.96	21.6	0.91	1.12
Ogden	0.34	18.1	0.81	1.08
Virginia Beach	0.70	21.0	1.05	1.20
Fresno	0.40	20.7	0.84	1.10
Baton Rouge	0.28	20.9	1.22	1.38
Detroit	1.87	24.5	0.94	1.13
Tulsa	0.42	19.6	0.96	1.17
