# Supplementary material for: Estimating road traffic impacts of commute mode shifts
Source: PLoS One. 2023 Jan 11;18(1):e0279738. doi: 10.1371/journal.pone.0279738 (PMC9833534; doi:10.1371/journal.pone.0279738)
Supplement: S5 Table — Result for all 74 analysed cites are shown. (PDF) [file pone.0279738.s005.pdf]

Table S5. Percent of adaptation to work-from-home needed to offset the influence of 25% and 50% transit shift to SOV, out of all potential SOV commuters. Result for all 74 analysed cites are shown.

	25% shift	50% shift
New York	17.22%	34.50%
San Francisco	10.88%	21.78%
Los Angeles	3.76%	7.61%
Boston	7.27%	14.35%
Philadelphia	5.03%	10.13%
Chicago	6.24%	12.49%
Seattle	6.30%	12.78%
Houston	2.63%	5.21%
Dallas	2.32%	4.60%
San Jose	3.80%	7.78%
Atlanta	3.05%	6.04%
Miami	3.55%	6.90%
Portland	4.24%	8.92%
Riverside	2.32%	4.90%
Orlando	2.57%	5.17%
Washington	7.53%	14.92%
Baltimore	3.47%	7.08%
Tampa	2.11%	4.58%
Denver	2.88%	6.08%
Providence	2.65%	5.01%
Jacksonville	3.01%	5.35%
San Diego	2.91%	5.93%
Phoenix	3.01%	6.19%
San Antonio	3.14%	5.90%
Cincinnati	2.52%	4.62%
Oxnard	1.77%	4.15%
Raleigh	1.33%	3.31%
Austin	2.69%	5.31%
St. Louis	1.61%	3.56%
Charlotte	2.36%	4.72%
Pittsburgh	3.53%	6.94%
North Port	0.69%	2.88%
Allentown	0.96%	3.32%
Oklahoma City	1.84%	3.77%
Nashville	1.86%	3.95%
Minneapolis	3.52%	6.78%
Charleston	2.38%	4.40%
Sacramento	2.59%	5.61%
Boise City	3.50%	5.57%
Santa Rosa	1.32%	4.03%
Kansas City	2.32%	4.12%
Savannah	3.96%	6.46%
Louisville	2.75%	4.98%
Reading	3.62%	6.37%
Bremerton	4.39%	9.32%
Richmond	3.26%	5.70%
Bridgeport	4.40%	9.48%
Buffalo	2.37%	4.77%
Boulder	6.03%	10.99%
Ann Arbor	3.39%	7.34%

Continued on the next page

Table S5 – continued from previous page

	25% shift	50% shift
Lexington	2.15%	4.34%
Omaha	2.25%	4.08%
Tucson	2.36%	5.72%
Durham	3.30%	6.55%
Colorado Springs	3.07%	5.30%
Lancaster	4.99%	8.17%
Hartford	1.70%	3.99%
Lincoln	5.34%	7.49%
Salinas	9.21%	15.13%
Rochester	6.41%	10.29%
Duluth	5.03%	7.45%
Salt Lake City	3.38%	6.84%
Provo	2.87%	6.03%
Greenville	1.55%	3.52%
Memphis	1.07%	2.83%
Stockton	3.22%	6.00%
Vallejo	6.54%	10.43%
Las Vegas	3.17%	6.55%
Ogden	3.39%	5.89%
Virginia Beach	2.81%	5.08%
Fresno	2.65%	5.53%
Baton Rouge	0.99%	3.00%
Detroit	2.36%	4.43%
Tulsa	2.55%	4.54%
