# Supplementary figures and images for: Estimating road traffic impacts of commute mode shifts
Source: PLoS One. 2023 Jan 11;18(1):e0279738. doi: 10.1371/journal.pone.0279738 (PMC9833534; doi:10.1371/journal.pone.0279738)

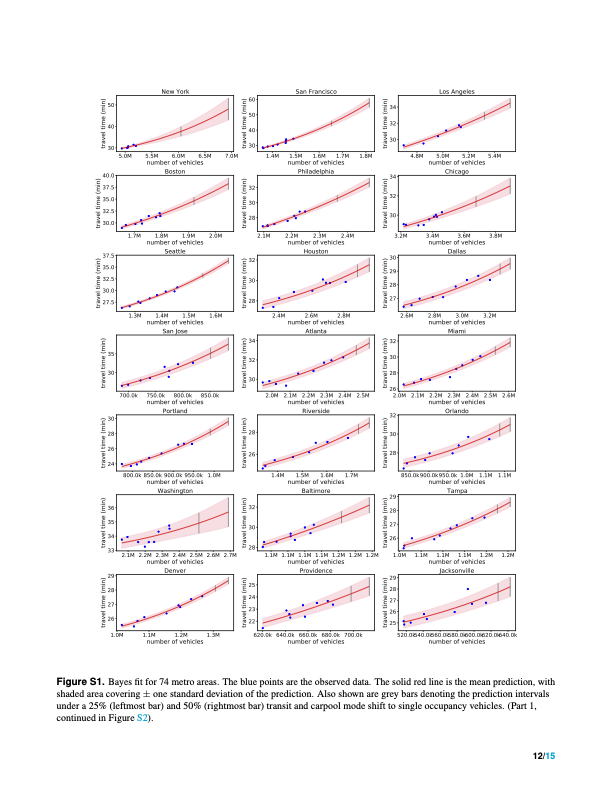

Supplement: S1 Fig — The blue points are the observed data. The solid red line is the mean prediction, with shaded area covering ± one standard deviation of the prediction. Also shown are grey bars denoting the prediction intervals under a 25% (leftmost bar) and 50% (rightmost bar) transit and carpool mode shift to single occupancy vehicles. (Part 1, continued in S2 Fig). (TIFF) [file pone.0279738.s006.tiff]

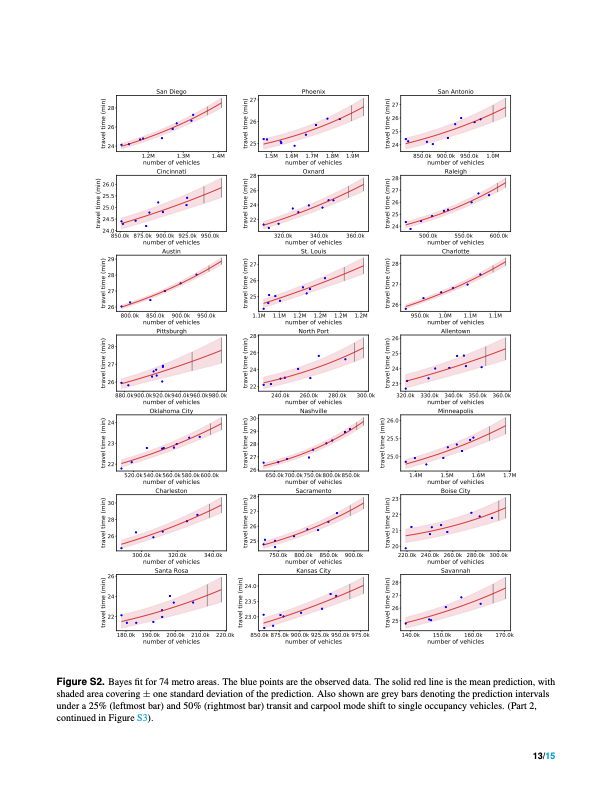

Supplement: S2 Fig — The blue points are the observed data. The solid red line is the mean prediction, with shaded area covering ± one standard deviation of the prediction. Also shown are grey bars denoting the prediction intervals under a 25% (leftmost bar) and 50% (rightmost bar) transit and carpool mode shift to single occupancy vehicles. (Part 2, continued in S3 Fig). (TIFF) [file pone.0279738.s007.tiff]

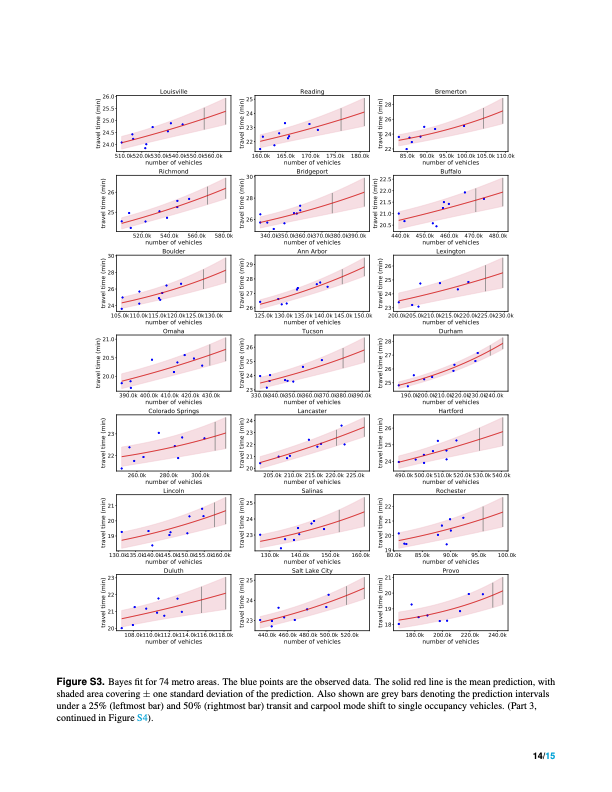

Supplement: S3 Fig — The blue points are the observed data. The solid red line is the mean prediction, with shaded area covering ± one standard deviation of the prediction. Also shown are grey bars denoting the prediction intervals under a 25% (leftmost bar) and 50% (rightmost bar) transit and carpool mode shift to single occupancy vehicles. (Part 3, continued in S4 Fig). (TIFF) [file pone.0279738.s008.tiff]

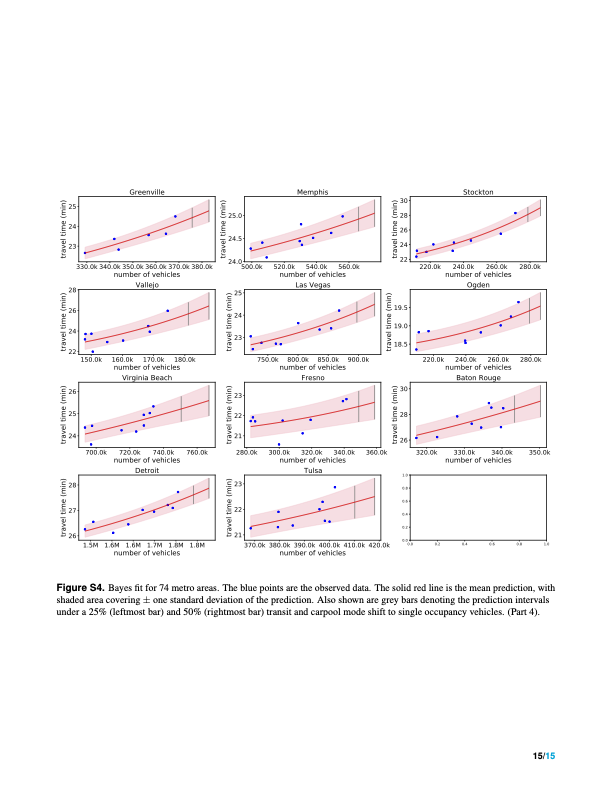

Supplement: S4 Fig — The blue points are the observed data. The solid red line is the mean prediction, with shaded area covering ± one standard deviation of the prediction. Also shown are grey bars denoting the prediction intervals under a 25% (leftmost bar) and 50% (rightmost bar) transit and carpool mode shift to single occupancy vehicles. (Part 4). (TIFF) [file pone.0279738.s009.tiff]
