# Supplementary material for: Estimating road traffic impacts of commute mode shifts
Source: PLoS One. 2023 Jan 11;18(1):e0279738. doi: 10.1371/journal.pone.0279738 (PMC9833534; doi:10.1371/journal.pone.0279738)
Supplement: Attachment — Submitted filename: rebuttal.pdf [file pone.0279738.s010.pdf]

Yue Hu, PhD candidate
Department of Computer Science,
Institute for Software Integrated Systems,
Vanderbilt University
1025 16th Ave S, Suite 102; Nashville, TN 37212

July 23, 2022

To the editorial board of the *PLOS One*:

We thank the Reviewers for the helpful comments and respond to the specific points in this letter.

Editor

We thank the editor for the opportunity to revise the manuscript in light of the questions and comments from the reviewers. The reviewers noted that our paper address an important problem of understanding the potential changes to traffic congestion if large scale mode shifts occur (reviewer 1), and that the paper is well written (reviewer 1 and 2). Both reviewers made suggestion on adding more to the literature review, which we did accordingly. The reviewers also raised questions on the modeling, figure style and paper structure, which we also checked and explained respectively.

Reviewer 1

Understanding the potential changes to traffic congestion if large scale mode shifts occur is important to maintain the efficient operation of road networks. In general, the paper is well written and structured. However, the reviewer have some questions, and I hope the reviewer can address them or provide a reasonable explanation. My major comments are below:

We thank the reviewer for the positive feedback, and address the major comments below.

1. The authors summarized the conclusion in the abstract, but what about the contribution in your study.

We have added the contribution statement in the abstract.

2. "We use the BPR model to describe the relationship between the number of passenger vehicles used for commuting and the corresponding average travel time". It may be incorrect. I think all of vehicles, not just for commuters should be considered here.

The reviewer is correct that we did not include all vehicles in the study. However, this is intentional. In this paper we regressed the commute time on the number of commuting vehicles. In this way, we provide a simple and elegant approach to estimate commute time based on information that we know, i.e., the number of passenger vehicles. The result shows that for most of the major metro areas in US, there is a strong correlation between commute travel time and number of passenger vehicles - details are reported in Section "Results", subsection "Data: American Community Survey commute data". Moreover, by using a Bayesian linear regression model, the uncertainty of the prediction, e.g., brought by not including all vehicles besides commuting cars, is measured. We finally note that the total number of vehicles is unknown in the dataset, so it is not possible to regress based on this quantity.

3. Some related works should be added and discussed. For example, Understanding vehicles commuting pattern based on license plate recognition data.

We thank the reviewer for pointing out the related works. They are added.

4. All of figures are ambiguous.

We double checked that the figures are properly referenced, and that the units and captions are complete. We would be happy to further modify the figures if we have omitted critical information.

Reviewer 2

The author has done a great job and there are 2 minor comments that I would like the author to revise.

First, the structure of the paper is similar to that of a comprehensive journal like nature communication, where the result is placed directly after the introduction. I have read other papers in plos one and most of them are not in this structure, so i suggest to adjust it.

We thank the reviewer for pointing out the potential manuscript organization concern. We note that according to the PLOS One guideline[1], our current organization of placing results directly after introduction is in line with the journal standards. Specifically, the guideline states that after beginning section (including abstract and introduction), in the middle section, the following elements can be renamed as needed and presented in any order: Materials and Methods, Results, Discussion, Conclusions (optional). There are also works in PLOS one that place results directly after introduction, like works [1, 2]. Therefore, we choose the current order for best readability.

Secondly, the latest reference is from 2020, which is relatively old, and there are fewer papers from transportation journals. I understand that the author's background is in computer science and has experience publishing in a top journal like TKDD. However, the topic of this paper is closely related to intelligent transportation systems (ITS), and the reviewers are in the field of ITS, so I suggest the authors cite at least three recent papers from journals in the field of ITS, e.g., Communications in Transportation Research and Journal of Intelligent and Connected Vehicles.

We agree with the reviewer that the reference is relatively old. We have added new publications on related topic in 2021 and 2022. Works in the ITS field, e.g. from Communications in Transportation Research and IEEE Transactions on Intelligent Transportation Systems are included.

Based on the comments and suggestions from the Reviewers, we have revised the manuscript. We look forward to receiving feedback on our revisions.

With kind regards,
Yue Hu

References

[1] Mehmet Yildirimoglu and Osman Kahraman. Searching for empirical evidence on traffic equilibrium. *PloS one*, 13(5):e0196997, 2018.

[2] Rohan L Aras, Nicholas T Ouellette, and Rishee K Jain. Automated identification of urban substructure for comparative analysis. *Plos one*, 16(1):e0245067, 2021.

[1] `https://journals.plos.org/plosone/s/submission-guidelines`
