# Supplementary material for: Estimating road traffic impacts of commute mode shifts
Source: PLoS One. 2023 Jan 11;18(1):e0279738. doi: 10.1371/journal.pone.0279738 (PMC9833534; doi:10.1371/journal.pone.0279738)
Supplement: Attachment — Submitted filename: rebuttal.pdf [file pone.0279738.s011.pdf]

Yue Hu, PhD candidate
Department of Computer Science,
Institute for Software Integrated Systems,
Vanderbilt University
1025 16th Ave S, Suite 102; Nashville, TN 37212

October 18, 2022

To the editorial board of the *PLOS One*:

We thank the Reviewers for the helpful comments and respond to the specific points in this letter.

Editor

We thank the editor for the decision to revise the manuscript in light of the questions and comments from the reviewers. We note that while our edits are accepted by the previous reviewer, there is an additional reviewer. We addressed his questions on modeling, literature reviews and term definitions accordingly.

Reviewer 1

The authors have addressed my comments well. Now, I recommend to accept the paper with the current version.

We thank the reviewer for the positive feedback.

Reviewer 3

This paper proposes a method to estimate the impact of commute mode shifts on commute travel time, which is an interesting and important topic. I mainly have the following comments:

Major suggestions:

1. I would like to know why the authors did the research only in MSA regions instead of modeling in all regions.

We choose to conduct the research on MSA regions, because the MSA regions have the most complete historical data available, provided by the U.S Census Bureau. Moreover, MSA covers regions with high population density, which are of most interest for our study on commuter pattern shifts.

2. I am not sure whether the parameter determination logic of the BPR model is correct. I think the free flow travel time and road network capacity should be determined through data or prior knowledge, and then fitted α and β of BPR model, rather than determine the α and β first, then get free flow travel time and road network capacity by fitting. And the determination of α and β requires more basis.

In the BPR model, the choice of $\alpha = 0.15$ and $\beta = 0.4$ is standard, and wide studies and applications have seen its applicability [1, 2, 3, 4]. In the mean time, the free flow travel time and the road network capacity across a whole region is hard to directly measure. Thus, we choose to fit the BPR model with fixed $\alpha = 0.15$ and $\beta = 0.4$, and derive the the free flow travel time and the road network capacity. We revised the manuscript to make this point more clear.

3. The data used to calibrate the free flow travel time and road network capacity of the city span about 10 years. In 10 years, the free flow travel time and road network capacity of the same city may change greatly, which needs to be taken into consideration.

The author is correct that there might be changes in free flow travel time and road network capacity for a city. Yet when we fit the historical data of 118 metro areas to the BPR model, we found that 74 of the metro areas have a Pearson correlation coefficient of larger than 0.5 and two tailed significance p value smaller than 0.1. This means that the influence of road network capacity shift is negligible for these 74 areas, and we restrict data fitting and analysis to the 74 metro areas in our work.

4. It is suggested to add a section of literature review, and give the methods used in similar researches and the advantages of this study.

Although we do not have a separate chapter on literature review, we have included the related works in the Introduction section. Specifically, works [5, 6, 7] analyze the daily vehicle commuting patterns in different ways, and works [8, 9, 10, 11] analyze the commute behavior change under COVID-19. And our work take one step forward and asks the important question: how will the shifts in commute patterns impact the road traffic? There are no previous works to answer this question, and our approach is unique.

1. The "universal PBR model" in line 26 in Introduction should be changed to "universal BPR model".

2. What does the "MSA" in line 49 in Results mean? No explanation is given. Is it the abbreviation of metropolitan statistical areas. The authors should check the full manuscript and explain any abbreviations and symbols before they first appear.

3. The explanations of τ and N4 in line 76 in Results are not given.

We thank the reviewer for pointing out the typo and lacking in definitions. We have fixed the manuscript accordingly.

Based on the comments and suggestions from the Reviewers, we have revised the manuscript. We look forward to receiving feedback on our revisions.

With kind regards,
Yue Hu

References

[1] Gary A Davis and Hui Xiong. Access to destinations: travel time estimation on arterials. 2007.

[2] Wai Wong and SC Wong. Network topological effects on the macroscopic bureau of public roads function. *Transportmetrica A: Transport Science*, 12(3):272–296, 2016.

[3] Rafał Kucharski and Arkadiusz Drabicki. Estimating macroscopic volume delay functions with the traffic density derived from measured speeds and flows. *Journal of Advanced Transportation*, 2017, 2017.

[4] Hui-fang Tan, Yang Yang, and Ling-rui Zhang. Improved bpr function to counter road impedance through od matrix estimation of freight transportation. *Journal of Highway and Transportation Research and Denelopment*, 11(2):97–102, 2017.

[5] Wenbin Yao, Maolei Zhang, Sheng Jin, and Dongfang Ma. Understanding vehicles commuting pattern based on license plate recognition data. *Transportation Research Part C: Emerging Technologies*, 128:103142, 2021.

[6] Xiaolei Ma, Congcong Liu, Huimin Wen, Yunpeng Wang, and Yao-Jan Wu. Understanding commuting patterns using transit smart card data. *Journal of Transport Geography*, 58:135–145, 2017.

[7] Kevin S Kung, Kael Greco, Stanislav Sobolevsky, and Carlo Ratti. Exploring universal patterns in human home-work commuting from mobile phone data. *PloS one*, 9(6):e96180, 2014.

[8] Ziheng Huang, Dujuan Wang, Yunqiang Yin, and Xiang Li. A spatiotemporal bidirectional attention-based ride-hailing demand prediction model: A case study in beijing during covid-19. *IEEE Transactions on Intelligent Transportation Systems*, 2021.

[9] Wenbin Yao, Jinqiang Yu, Ying Yang, Nuo Chen, Sheng Jin, Youwei Hu, and Congcong Bai. Understanding travel behavior adjustment under covid-19. *Communications in Transportation Research*, page 100068, 2022.

[10] Shaila Jamal, Sadia Chowdhury, and K Bruce Newbold. Transport preferences and dilemmas in the post-lockdown (covid-19) period: Findings from a qualitative study of young commuters in dhaka, bangladesh. *Case studies on transport policy*, 10(1):406–416, 2022.

[11] Samuele Marinello, Francesco Lolli, and Rita Gamberini. The impact of the covid-19 emergency on local vehicular traffic and its consequences for the environment: The case of the city of reggio emilia (italy). *Sustainability*, 13(1):118, 2020.
